# Child and Adolescent Mental Health Service (CAMHS), Terengganu, Malaysia: milestones so far and the paths to the future

**DOI:** 10.1080/17571472.2018.1484318

**Published:** 2018-06-18

**Authors:** Rahima Dahlan, Mohd Nizam Abd Ghani, Rosliza Yahaya, Tuan Sharipah Tuan Hadi

**Affiliations:** a Faculty of Medicine, University Sultan Zainal Abidin, Kuala Terengganu, Malaysia; b Department of Psychiatry and Mental Health, Hospital Sultanah NurZahirah, Ministry of Health, Kuala Terengganu, Malaysia

**Keywords:** Child, adolescent, mental-health-service, Malaysia, Terengganu

## Abstract

This study aims to provide an overview of mental health problems of children and adolescents in Malaysia in general and the state of Terengganu in particular. It also highlights the challenges and the opportunities in the establishment of child and adolescent mental health services (CAMHS). CAMHS in Malaysia are developing slowly but have not reached the standards found in developed countries. Significant improvements are needed to ensure that the service can provide optimal help to children and adolescent as well as their families. Constraining factors such as a lack of trained workers, limited financial resources for training and inadequate facilities are among the challenges. Despite all these challenges, specific strategies are required to optimally utilise the potential existing resources. The Ministry of Health initiatives in creating and implementing the national mental health policy and increasing mental health awareness campaigns for children and adolescents are of paramount importance. To overcome the lack of resources in the implementation of CAMH services, in-service education and training, integration of mental health services with the existing primary health care facilities and cultivation of cooperative and communicative networks between primary care professionals, mental health workers and other relevant agencies are crucial steps.

**Why this matters to me**

We describe an example of CAMHS in Terengganu, Malaysia to illustrate the importance of strategic planning in developing a service in a resource-constrained environment. Inadequate funds for training and a lack of health professionals needed to form multidisciplinary teams are among the greatest challenges during the initial phase of CAMHS implementation. CAMHS Terengganu was initiated in December 2004 and is led by a child and adolescent psychiatrist along with three other health workers to provide services to children and adolescent and their families in Terengganu and the east coast. Over the years, efforts were made to improve knowledge and to provide necessary skills through continuous in-service education and training among mental health practitioners and primary care professionals. The aim was to consolidate existing knowledge and employ evidence-based approaches for better skills in assessing and managing children and adolescent with mental health problems. The implementation of CAMHS in Terengganu has served to facilitate numerous improvements in promoting the prevention and increasing awareness of mental health problems in young people. The authors of this paper are keen to share their experience with others who are responsible for developing CAMHS. It matters to us greatly because it is impossible to deliver good quality CAMHS without understanding the possible challenges and realising opportunities.

**Key message**

Government strategy is the key element of developing CAMH service in a resource-constrained environment.

## Socio-demographics: National and local health context

Malaysia is an upper-middle income country [1] with a population of 28.7 million citizens [2]. Demographically, it is a diverse, multiracial country consisting of Bumiputeras (predominantly Malays in Peninsular Malaysia, Ibans and Kadazan/Dusun in Sarawak and Sabah respectively), Chinese, Indians and others. 69.7% of the country’s population is aged between 15–64 years, 24% under 14 years of age and 6% above 65 years. The life expectancy is 72 years for males and 77 years for females [2].

Terengganu has a population of 1.2 million. It is one of the states with 8 districts located on the East coast of Malaysia. The life expectancy is lower than the national rate, namely, age 68 years for men and 74 years for women [2]. National and health context of Malaysia are summarised in Table 1.

**Table 1.** Malaysia: National and local context at a glance.

National (Malaysia)	
*Population of citizen (million):	28.7
• Male	14.5
• Female	14.2
*By age group (years):	
• 0–14	7.7 (24.1%)
• 15–64	22.3 (69.7%)
• 65+	2.0 (6.2%)
*Life expectancy at birth (years)	
• Males	72.7
• Females	77.4
**World bank income group (2010)	Upper middle income
**Gross national income per capita (PPP Int. $, 2013)	22
**Total expenditure on health per capita (PPP int. $, 2014)	1,040
**Total expenditure on health as a percentage of GDP (2014)	4.2
Local (Terengganu)	
*Population (million)	1.21
• Male	0.62
• Female	0.59
*Life expectancy (years)	
• Male	68.8
• Female	74.6

* Source: Selected demographic statistics estimates, Malaysia 2017 (https://www.dosm.gov.my/v1/index.php), assessed on 26 March 2018.

** Global Health Observatory World Health Organization (WHO)|Malaysia, (http://www.who.int/countries/mys/en/), assessed on 26 March 2018.

## Mental health statistics

Mental health problems in children are a growing concern due to their wide-ranging effects on self-esteem, behaviour, emotional development, educational attainment, social relationship and quality of life. British Nationwide survey [3] indicated 1 in 10 of children and young people below age 16 years had a diagnosable mental health disorder and less than half had any contact with mental health workers [3]. The most common disorders were disruptive behaviours such as oppositional defiant disorder and conduct disorders, emotional disorders (anxiety and depression) and attention deficit hyperactivity disorder [3]. In Asian countries, the prevalence of children and adolescents with mental health disorder ranges from 10 to 20 percent [4].

In Malaysia, the national-level data from National Health & Morbidity Survey (NHMS) revealed an upward trend of mental health problems among children aged 5–15 years from 1996 (13.0%), 2006 (19.4%) and 2011 (20.0%) [4], but showed a reduction in overall prevalence in 2015 (12.1%) [4]. The decline in prevalence is likely due to a variety of strategies being carried out by the Ministry of Health (MOH), other ministries and non-government agencies which involve a change in policy as well as a restructuring of the health system in the country. For example, since 2000 integrated management of mental health problems at the primary health care level has been developing [5]. Early detection and intervention program for Autism Spectrum Disorder in the primary health care setting has also started, with a validated screening questionnaire to improve detection. Also, the implementation of the special education system by the Ministry of Education since the year 2001 aimed at meeting the needs of special needs children might have contributed to the reduction in mental health problems among this group. Other relevant strategies include active participation and contribution from all health professionals working in the mental health sectors and public health in developing clinical guidelines, protocols and training modules on child and adolescent mental health. The role of non-governmental agencies in promoting and increasing public awareness and mental health advocacy is also one of the crucial steps in the provision of better mental health service.

There are significant ethnic differences in the prevalence of mental health problems in Malaysia, being highest in other Bumiputeras (i.e. Sarawak and Sabah) (16.5%) and lowest in Malays (10.4%) [4]. As demonstrated in Table 2, peer-related difficulties (32.5%) and conduct problems (16.7%) are the most prevalent mental health issues, followed by emotional problems (15.7%) and hyperactivity (4.6%) [4]. The recent NHMS 2015 survey also found that boys, younger children (5–9 years) and those living in rural areas were at high risk of mental health problems [6]. Given these vulnerabilities to mental health problems, there is a particular need to create a mental health service focused on children and adolescents who are socio-economically disadvantaged [4].

**Table 2.** Median rate of human resources per 100,000 population working in the mental health sector by World Bank income group.

Income group (World bank)	Psychiatrist	Other medical doctors	Nurses	Psychologists	Social workers	Occupational therapists
Malaysia	0.83	UN	3.31	0.29	UN	UN
Upper middle income countries	2.03	0.87	9.72	1.47	0.76	0.23
World	1.27	0.33	4.95	0.33	0.24	0.06

Note: UN = Information unavailable.

Source: Mental Health Atlas 2011 – WHO website http://apps.who.int, assessed on 17 May 2018.

## Policy for child mental health

The National Mental Health Service Policy (NMHSP) framework [7] has been the blueprint for mental health service in Malaysia since 2001. The vision set out in the framework emphasises the promotion of mental health and prevention of psychological problems. Aligned with the MOH goals, NMHSP primarily aims to provide service to those with the greatest overall needs through comprehensive care, treatment, control, protection and rehabilitation [7].

The integration of mental health care into primary health care systems under the public health administration [7] has cultivated cooperative and communicative networks between primary care professionals and is crucial for effective service linkage. This network has led to better mental health awareness in the community and further improvement in the care of patients with mental health illnesses. The scope of service includes mental health promotion, early detection and intervention (individual and family), follow-up of mentally-ill patients and psychosocial rehabilitation [7]. Cultivating cooperative and communicative networks between primary care professionals is crucial for effective service network.

In Malaysia, mental health services for children and adolescents were initially started by general psychiatrists. The Mental Health Atlas 2011 [8] by the World Health Organisation (WHO) has revealed a considerable difference in median numbers of health professionals in the mental health sector in Malaysia compared with other upper-middle-income countries (Table 2) [8,9]. There are 0.83 psychiatrists per 100 000 population, significantly fewer than in other upper-middle-income countries.

There is also a very significant shortage of trained child and adolescent mental health practitioners not only in Terengganu but in the whole of Malaysia. At present, approximately 20 child psychiatrists are available throughout the country, and they are mainly distributed in the urban areas. Malaysian services still lack clinical psychologists, psychotherapists, occupational therapists and speech and language therapists required to form multidisciplinary teams [7]. Despite such constraints, Malaysian professionals have endeavoured to deliver services that are readily and widely available; continuous efforts have been made in improving knowledge and skills of paediatricians and family physicians in the management of less complicated child and adolescent patients with mental health disorders in their settings.

## The specific local context - CAMHS in Terengganu, Malaysia

The prevalence of mental health problems among children in Malaysia and Terengganu in the year of 2006 [10] and 2015 [6] is illustrated in Table 3. The NHMS in the year 2015 [6] shows an overall prevalence of mental health problems among children and adolescent in Terengganu to be lower (9.9%) than the national prevalence (12.1%). However, the prevalence of peer-related problems is higher (36.9%) and pro-social skills are lower (8.1%) when compared with the national figures (Table 3) [6].

**Table 3.** Prevalence of mental health problems among children 5–15 years in Terengganu in comparison with national statistics in the NHMS 2006 and 2015.

NHMS	Prevalence (%)	
	Overall	
	National (Malaysia)	Terenganu
^a^2006	20.3	26.6
^b^2015	12.1	9.9
^b^SDQ domains in NHMS 2015		
Emotional problems	15.7	12.0
Conduct problems	16.7	13.6
Hyperactivity	4.6	4.0
Peer problems	32.5	36.9
Prosocial skills	11.2	8.1

Note: Questionnaire used in NHMS.

a Reporting Questionnaire for children (RQC).

b Strength and Difficulty Questionnaire (SDQ).

In December 2004, a child psychiatry clinic was re-established in Terengganu and re-branded with the new name of Child and Adolescent Mental Health Service (CAMHS). The CAMHS was managed with a team consisting of one consultant child and adolescent psychiatrist, one medical officer who was attached to this clinic on a rotational basis for a year, an assistant nurse and an attendant. The main service provided was screening, diagnosing and interventions by the team.

**Figure 1.**
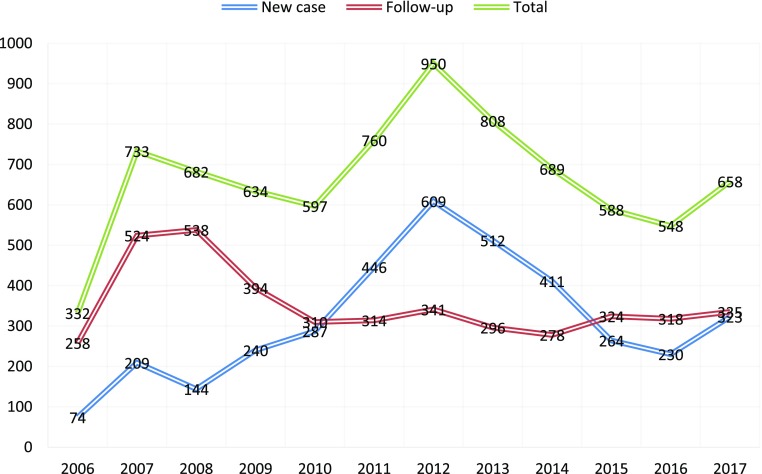
CAMHS: number of patients, 2006 to 2017.

As shown in Figure 1, the number of patients in the year 2006 was low as it was the first year of the introduction of CAMH service with a limited input from the child and adolescent psychiatrist. In 2012, the number of patients seen had increased dramatically in line with the increase of the input of the psychiatrist. The geographical distribution of the referrals had increased significantly as well. The slight decline in the number of treated patients in subsequent years might be attributed to the on-going training provided by the CAMHS team to other health workers, especially in district hospitals and primary health care.

**Figure 2.**
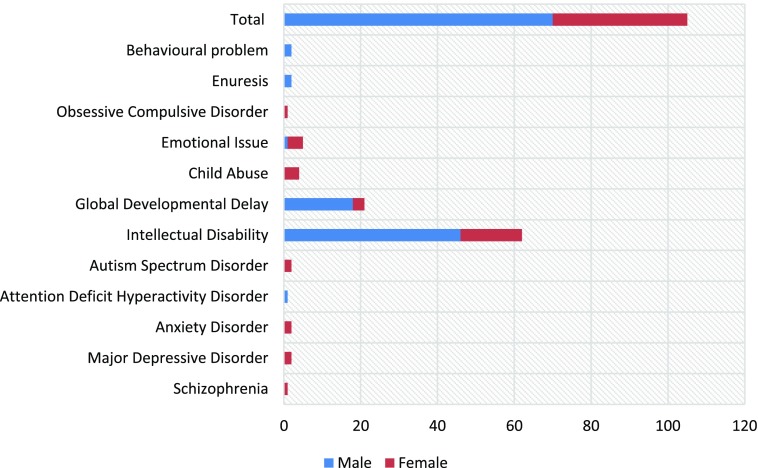
Diagnosis of new patients in 2017.

Latest figures indicate that the most frequent diagnoses in 2017 were Intellectual Disability (59%) and Global Developmental Delay (20%), while diagnoses with the lowest frequency (0.9%) were Attention Deficit Hyperactivity Disorder, Schizophrenia and Obsessive Compulsive Disorder (Figure 2). It is important to note that the high frequency of the Intellectual Disability diagnoses was due to the inclusion of specific learning disorders into this category. The two conditions will be separated in the future.

Despite having a lower overall prevalence compared with national figure, the number of children with mental health problems in Terengganu remains a concern. Particular emphasis should be given to the reduction of mental health problems associated with peer-related difficulties and to creating programmes or activities with a focus on improving pro-social skills and appropriate life-skills. There is still an urgent need to strengthen, refine and upgrade the existing CAMH service in Terengganu.

## Disclosure statement

No potential conflict of interest was reported by the authors.

## Governance

National Institutes of Health (NIH), Ministry of Health (MOH), Malaysia oversaw this work.
